# Uptake and accumulation mechanisms of hexachloroplatinate(IV) ions in the unicellular alga, *Pseudococcomyxa simplex*

**DOI:** 10.1093/mtomcs/mfae009

**Published:** 2024-01-31

**Authors:** Masato Tokoro, Yu Imamura, Kazuhiro Kumagai, Akiko Hokura

**Affiliations:** Graduate School of Engineering, Tokyo Denki University, 5 Senju-Asahicho, Adachi, Tokyo 120-8551, Japan; Graduate School of Engineering, Tokyo Denki University, 5 Senju-Asahicho, Adachi, Tokyo 120-8551, Japan; Nanodimensional Standards Group, Research Institute for Material and Chemical Measurement National Metrology Institute of Japan (NMIJ), National Institute of Advanced Industrial Science and Technology (AIST), Tsukuba Central 5, 1-1-1 Higashi Tsukuba, Ibaraki 305-8565; Department of Applied Chemistry, School of Engineering, Tokyo Denki University, 5 Senju-Asahicho, Adachi, Tokyo 120-8551, Japan

## Abstract

Platinum uptake was examined by adding hexachloroplatinate(IV) solution to the unicellular alga *Pseudococcomyxa simplex*. After the addition of platinum solution ([Pt] = 100 mg/kg, pH 3.2–3.4) for a certain time, the cells were quickly frozen and subjected to μ-XRF (X-ray fluorescence) analysis using synchrotron X-rays. The beam size of approximately 1 micrometer allowed visualization of the platinum distribution within a single cell. On the other hand, we examined platinum uptake in enzyme-treated protoplasts and lyophilized cells and found that the platinum uptake concentrations in these samples were higher than in living *in-vivo* cells. Cell wall and cell metabolism were presumed to interfere with the uptake of hexachloroplatinate(IV) ions. All platinum ions taken up by the cells were reduced to divalent form. The effect of light on platinum addition was also investigated. When platinum was added under light conditions, some samples showed higher platinum accumulation than under shade conditions.

## Graphical Abstract

**Graphical Abstract:**
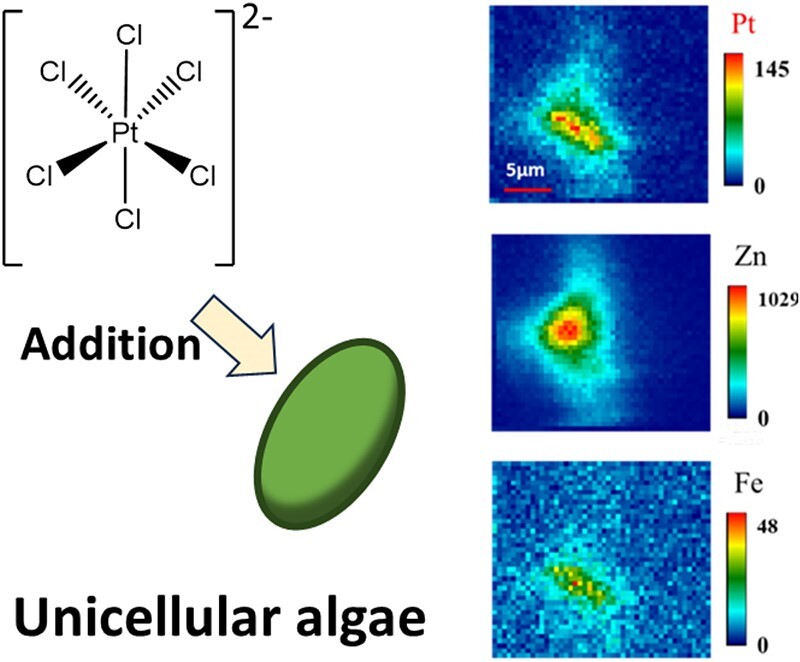
Hexachloroplatinate(IV) was added to unicellular algae and its uptake into the cells was visualized. In the cells, platinum was reduced to divalent and its ligands were N or O chemical species.

## Introduction

Plants that accumulate high concentrations of heavy metals are called hyperaccumulators and are expected to be applied to remediation technology for toxic heavy metal contamination and recovery technology for useful metals.^1,2^ For example, lead and gold have been successfully recovered from industrial wastewater by using protonemata of moss *Funaria hygrometrica*.^3^ There has also been interest in the chemical forms of heavy metals incorporated into plants: gold nanoparticles were formed intracellularly when tetrachloroauric(III) acid solution was added to microalga *Chlorella vulgaris*,^4^ silver nanoparticles were formed when silver(I) ions were added to algae or their extracts^5,6^ and metallic tellurium nanorods were formed when telluride or tellurate was added to algae,^7,8^ etc. Thus, research on the production of metal nanoparticles using the functions of plants, especially microalgae, has been progressing rapidly in recent years.^9^

Metallic platinum is used industrially in various fields because it is chemically very stable, resistant to acids and alkalis, heat resistant due to its high melting point, and has excellent catalytic properties.^10^ Although platinum has a low ionization tendency, it takes on the +2 and +4 oxidation states as halide complexes and oxides. Cisplatin, a divalent platinum complex, is used as an anticancer drug, and the cellular uptake and metabolic mechanisms of cisplatin and similar compounds have attracted much attention.^11^

It has been reported that microalgae accumulate platinum when platinum complexes were added. The marine microalgae *Chlorella stigmatophora* accumulated as high as 30 mg/kg of platinum. It was shown that the accumulation rate of platinum was much slower than that of palladium and rhodium, which are also members of the platinum group, and that the rate of platinum uptake in the algae was constant at 20% even when the concentration of added platinum was changed.^12^ Furthermore, it was reported that lyophilized cells of *Galdieria sulphuraria*, a sulfidic hot-spring red alga inhabiting hot and acidic conditions, recovered high levels of platinum. The chemical form of platinum recovered was not metal or oxide nanoparticles, but a sulfur-coordinated platinum complex, [(C_2_H_5_)_2_S]PtCl_2_.^13^

Thus, the accumulation of platinum in microalgae has been reported, but the details of the accumulation mechanism remain unclear. To practically implement platinum recovery using algae, specificity for platinum is crucial. Metal specificity has been reported in several algae,^14–16^ and finding such specificity is important for metal recovery and bio-mining applications. To understand the metal specificity of algae, it is crucial to determine their accumulation mechanism. To elucidate this accumulation mechanism, it is necessary to clarify the changes in the chemical form of platinum upon uptake and the intracellular accumulation sites.

The purpose of this study is to gain insight into the cellular uptake and accumulation of platinum by the addition of hexachloroplatinate(IV) to the unicellular alga *Pseudococcomyxa simplex*. This alga is an aerobic, freshwater-grown cell that is easily grown in the laboratory. In this study, we attempted to visualize intracellular platinum distribution by micro-XRF (X-ray fluorescence) imaging with minimal sample preparation and with the cells as alive as possible. Using a high-brilliance synchrotron radiation X-ray microbeam, the distribution of platinum in an *in-vivo* single cell of approximately 5 μm was revealed for the first time. In addition to living algal cells, protoplast samples with cell walls removed by enzymatic treatment, lyophilized cell samples, and acetone-extraction residue samples were prepared, and platinum uptake was examined by adding hexachloroplatinate(IV) solution to each. The effect of light irradiation on platinum accumulation was also examined. X-ray absorption spectroscopy was used to determine the chemical form of platinum incorporated into the algal cells.

## Methods

### Culture of the unicellular alga *P. simplex*

The unicellular alga *P. simplex* (NIES Collection NIES-2713, National Institute for Microbial System Conservation, National Institute for Environmental Studies) was used. Cultures were conducted in 1000 ml Marine flasks (BMS-MF1000) using the liquid suspension culture method at room temperature (22–26°C) and light intensity of 3000–6000 lux.^17^ Subcultures were performed every 2 weeks. The composition of the culture medium is shown in Table S1 in Supplementary data.

### Preparation of varied algal samples (*in vivo*, protoplasts, soluble component, lyophilized, and acetone-treated residue)

**Fig. 1:**
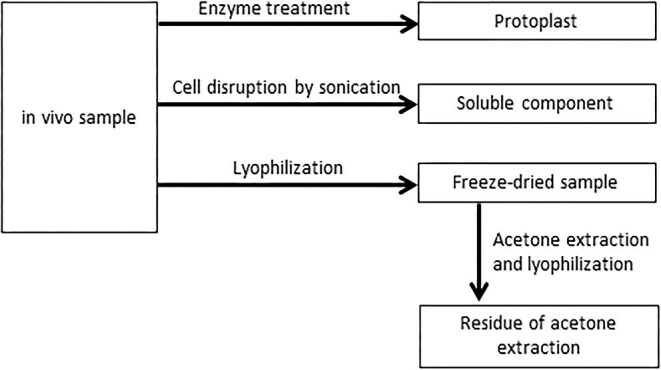
Alga samples prepared.

A volume of 30 ml of cultured single-cell algae were aliquoted into 50 ml conical tubes and centrifuged (8000 rpm, 3 min, 20°C) in a centrifuge (HITACHI CF15RXII) to remove the supernatant culture medium, and the collected cells were used as living cellular samples. To examine platinum uptake in algae, varied samples were prepared as shown in Fig. 1.

Protoplast samples were obtained by adding an enzyme solution to the algal cells and removing the cell walls as follows. Enzyme solution (hereafter referred to as enzyme solution A, pH 7.35) was prepared to be 0.6 M mannitol, 0.1% pectolyase Y-23, and 1% cellulase Onozuka RS.^18^ Single-celled algae were collected from 20 ml of the culture medium, washed with pure water, and 20 ml of enzyme solution A was added and allowed to stand in the dark for 2 days. Then, the protoplast samples were then collected by washing twice with 0.6 M mannitol.

Algal soluble component samples were prepared as follows: 5 ml of algal solution was placed in a tube with the entire culture medium, and the cells were crushed by an ultrasonic crusher (QSONICA Q125 micro-ultrasonic homogenizer). To prevent overheating of the sample, the tube was cooled, while disruption was performed at 30-second intervals for 10 minutes at a 30/30 pulse (50% power). Then, the disrupted sample was placed in a conical vessel and centrifuged (8000 rpm, 3 min) twice to remove the residue. The supernatant was collected, and soluble components were recovered. An optical microscope was used to confirm that no cells remained in the solution.

Lyophilized samples were prepared as follows: the living cellular samples were freeze-dried for 24 hours, ground, and homogenized in an agate mortar to make lyophilized samples.

Acetone extraction residue samples were prepared as follows. A fixed amount of the lyophilized sample was placed in a conical tube, and 30 ml of 80% acetone was added per 0.1 g of the lyophilized sample and shaken for 5–7 days to extract and remove chlorophyll and lipids. The residue was washed twice with deionized water, lyophilized, and powdered. This was used as the acetone-treated residue sample.

### Preparation of platinum-added solution

A 1000 mg/kg platinum standard solution (chemical form H_2_PtCl_6_, 5% HCl, Wako 165-28341) for inductively coupled plasma spectrometry (ICP) analysis was diluted with pure water and 2 M NaOH was used to adjust the pH of the solution to 3.2–3.3. The platinum concentration was set to 100 mg/kg (ppm).

### Addition of platinum(IV) solution to live cellular samples and protoplast samples

To the aliquoted live algal cells, 30 ml of H_2_PtCl_6_ solution (platinum concentration was 100 ppm) was added. The amount of algal cells was approximately 0.1 g in dry mass. The algae with the platinum solution were shaken (100 rpm) for a certain time in an incubator (TOMY CLE-303) using a horizontal shaker (TAITEC NR-3). The incubator was set at 27.0°C (day 16 hours) and 23.0°C (night 8 hours), humidity of 70–80%, and light intensity 12 000 lux (day 16 hours) and 400 lux (night 8 hours). For protoplasts, 20 ml of platinum solution at 100 ppm concentration was added and shaken in the incubator for a certain time. In both cases, light-shielded samples were also prepared with aluminum foil and subjected to the same experiments. The experiment was conducted in an incubator under constant temperature conditions, and light irradiation did not cause any temperature change.

### Addition of platinum(IV) solution to soluble component samples

A volume of 3 ml of platinum solution was added to 3 ml of soluble-component fraction and shaken in the incubator for a certain time. A light-shielded sample was also prepared in the same way.

### Addition of platinum(IV) solution to lyophilized samples, acetone-treated residue samples, and cellulose powder

The following procedures were carried out using lyophilized samples and acetone-treated residue samples and commercial cellulose powder (Wako 036-22225). An amount of 0.1 g of the dried mass was placed in a conical tube to which 30 ml of platinum solution was added and shaken in the incubator for a certain time. A light-shielded sample was also prepared with aluminum foil and tested in the same way.

### Sample preparations for X-ray fluorescence analysis and X-ray absorption spectroscopy

Algal samples (live cell samples, lyophilized samples, acetone-treated residue samples, protoplast samples, and cellulose powder) shaken with the platinum solution for a certain time were collected by centrifugation. Each sample was then washed twice with deionized water, freeze-dried for 24 hours, and ground and homogenized using an agate mortar. Ten milligram of each was weighed and tablets of 5 mm diameter were obtained by compression molding. These tablet samples were subjected to XRF and X-ray absorption fine structure (XAFS) analyses. For liquid samples, small volumes (approximately 3 ml) were wrapped in a polymer film (Mylar film^®^) and subjected to XAFS analysis. Alternatively, they were soaked in filter paper and then wrapped in polymer film for X-ray analysis.

### Sample preparation for μ-XRF imaging

**Fig. 2:**
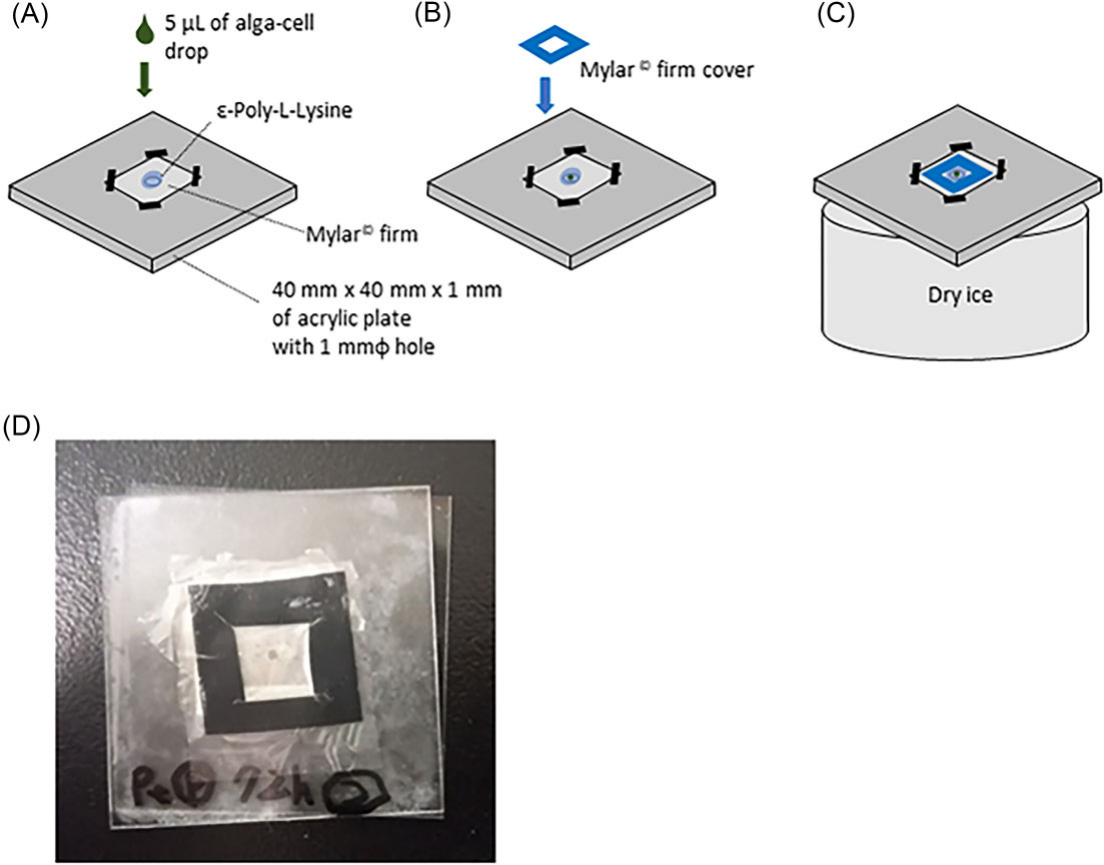
Sample preparation for micro-XRF imaging. (A) On top of the ε-poly-L-lysine-coated polymer membrane, 5 μl of a solution containing algal cells was dropped. (B) A polymer membrane cover was placed over it. (C) The sample was quickly frozen on dry ice. (D) A photograph of sample prepared.

Algal samples (live cell samples and lyophilized samples) that had been shaken with the platinum solution for a certain time were collected by centrifugation and washed twice with deionized water. The following procedures were then carried out for μ-XRF imaging, as shown in Fig. 2. An acrylic plate (40 mm x 40 mm x 1 mm) with a 1 mm hole in the center was used as the sample plate for μ-XRF imaging. A polymer film was taped over the hole. Since the polymer membrane is highly hydrophobic, α-polylysine (Sigma–Aldrich P8920-100ML) was applied to the surface of the polymer membrane. α-Polylysine is used as a surface coating agent for plastic culture vessels to improve the adhesion of cultured cells. After applying α-polylysine to the polymer membrane for 30 minutes, the excess polylysine solution was removed by absorbing it with filter paper. Next, 5 μl of a solution containing algal cells was dropped onto the polymer membrane, covered with a polymer membrane cap, and flash-frozen with dry ice and liquid nitrogen. Samples prepared in this way were used for μ-XRF imaging. This sample preparation is our original method, optimized to measure algal cells in as viable a state as possible.

### Measurement of platinum accumulation in algae by XRF analysis

A three-dimensional energy-dispersive X-ray fluorescence spectrometer with polarized optics (Epsilon 5, PANalytical) was used to measure platinum accumulated in algae. The platinum concentration was calculated using the calibration curve method. For the calibration curves, platinum oxide (PtO_2_, Wako, 169-08341) and cellulose powder were mixed as appropriate, and samples with platinum concentrations ranging from 0 to 100 000 ppm were used. The XRF measurement conditions were set as follows: the voltage and current of the X-ray tube (Gd anode) were 80 kV and 6 mA, respectively; zirconium was used as the secondary target material; and the measurement time for one sample was 300 seconds. The X-ray fluorescence intensity area of the platinum Lα peak (9.4 keV) was obtained in the algal samples. The mean value of *n* = 3 samples was calculated as the platinum accumulation concentration.

### Elemental distribution analysis of a single algal cell by μ-XRF imaging

Two-dimensional μ-XRF imaging was performed at BL37XU in SPring-8 (Hyogo, Japan). The beam from the undulator was monochromatized at 12.5 or 10.0 keV by a Si(111) double-crystal monochromator. The incident X-ray was focused by Kirkpatrick–Beaz mirror system to the size of 0.8 μm (vertical, V) × 1.0 μm (horizontal, H) on the sample surface. The sample was spatially scanned in steps of 0.5 μm (V) × 0.5 μm (H) by using stepping motors. The fluorescence X-rays from the sample were collected by a Si(Li)-solid state detector placed at 90° to the incident X-ray beam and integrated for 2 seconds at each point. To keep the samples frozen during the several hours of measurements, the samples were exposed to a cryogenic nitrogen gas stream (Cryojet XL, Oxford Instruments, UK).^19^ Data analysis was performed in LabVIEW 7.0 (National Instruments). Among the XRF spectra obtained at the measurement points, the XRF intensity in the ROI (Region of Interest) of a specific element was calculated and displayed on a 256-gradation color scale, with red denoting the highest intensities and blue the lowest.

### Chemical speciation of platinum accumulated in algal samples

The platinum L_3_-edge (11,585 eV) X-ray absorption spectrum was measured at beamline 12C at Photon Factory, the High Energy Accelerator Research Organization (Tsukuba, Japan). Synchrotron radiation X-ray from a bending magnet was monochromatized with a double-crystal monochromator. A bent cylindrical mirror was used as the focusing optics. The beam size was approximately 1 mm square.^20^ For the transmission method, the incident intensity, *I*_0_, and the transparent intensity, *I*, were measured using ionization chambers filled with N_2_(85%)–Ar(15%) and N_2_(50%)–Ar(50%), respectively. For the fluorescence method, a 19-element germanium solid-state detector was used, and a gallium filter (absorption edge of 10.368 keV) and a Soller slit were placed in front of the detector to reduce the scattering X-rays, thus improving the signal-to-background ratio of the measurements. Energy calibration was performed using a platinum foil, and the maximum peak was adjusted to 11 562 eV.^21^

Some platinum compounds with known chemical forms, such as platinum foil (metallic platinum), PtCl_2_, cisplatin (*cis*-diamminedichloro-platinum(II)), PtO_2_, and H_2_PtCl_6_, were prepared as reference materials. These reference materials were prepared by diluting them with boron nitride as appropriate and measured by the transmission method. The solution of H_2_PtCl_6_ was also subjected to XAFS analysis.

The XAFS analysis was performed using a program of REX2000 Ver.2.5 (Rigaku, Japan). The X-ray absorption near edge structure (XANES) spectra of the L_3_ absorption edge of platinum are sensitive to the 5d electron number of Pt atom and therefore useful for estimating the oxidation number and the coordination environment. Here, the Pt L_3_ spectra in the energy range 11 550 to 11 600 eV were used in the analysis with reference to the previous report.^22^ The XANES spectral intensities were normalized by the intensity at 11 585 eV and the intensity at 11 566 eV was read as the white line intensity.

The χ(k) spectra were weighted by *k*^3^ and then computed as Fourier transformed (FT) using the window function. The phase shift was not corrected in the FT. *Ab initio* theoretical amplitude and extended-phase functions were calculated using the McKale curved wave theory tables and were then used for the curve-fitting factor (%) calculated as


\begin{equation*}
{R}^2 = \ \frac{{\sum {{\left\{ {{k}^3{\chi }_{obs}\left( k \right) - {k}^3{\chi }_{calc}\left( k \right)} \right\}}}^2}} {{\sum {{\left\{ {{k}^3{\chi }_{obs}\left( k \right)} \right\}}}^2}} \times 100.
\end{equation*}

## Results and discussion

### Platinum concentrations accumulated in algal samples prepared by different methods

Table 1 shows the platinum concentrations accumulated in algal samples prepared by different methods when hexachloroplatinate(IV) solution was added to each sample for a given time. The platinum concentrations were determined by X-ray fluorescence analysis. Prepared algal samples include *in vivo* samples (living cells), protoplast samples, lyophilized samples, and acetone-extracted residue samples. The platinum concentrations of each sample taken up at a certain time are shown. Table 1 also shows the results under light-exposed and light-shielded experimental conditions.

**Table 1:** The platinum concentration of algal samples with platinum under various conditions

	Pt concentration (ppm)						
		Addition time (hours)					
Sample conditions	Lighting condition	1	6	24	48	72	168
*in vivo*	Lighted	860	1060	1410	2340	2870	5040
	Shielded	810	960	1060	1400	1500	2240
Protoplast	Lighted					5230	
	Shielded					4820	
Lyophilized	Lighted	3010		8070		14 360	
	Shielded	2370		4930		8600	
Acetone-treated residue	Lighted			9490		15 870	19 540
	Shielded			2780		5610	

After 72 hours of platinum solution addition, the *in vivo* samples accumulated 1500 ppm (shading) and 2870 ppm (lighting) of Pt. After 168 hours (7 days), 2240 ppm (shading), and 5040 ppm (lighting) Pt accumulated more than twice as much under the lighting condition.

Protoplast samples prepared by removing the cell wall accumulated 4820 ppm (shading) and 5230 ppm (lighting) of Pt at 72 hours (3 days) after the addition. In protoplasts, there was no significant difference in Pt accumulation concentrations between lighting and shading conditions. Protoplasts showed two to three times higher Pt accumulation than living *in vivo* samples without any treatment.

The lyophilized samples with platinum solution accumulated 8600 ppm (shading) and 14 360 ppm (lighting) of Pt after 72 hours, which was approximately five times the accumulation concentration in the living samples.

The acetone-treated residue samples also accumulated 5610 ppm (shading) and 15 870 ppm (lighting) of Pt after 72 hours of addition, two to three times the concentrations in the *in vivo* samples. This acetone-treated residue sample was a residue from which many components have been removed, including soluble components, chlorophyll, and lipids. Therefore, samples of commercially available cellulose with platinum were prepared and compared in terms of platinum accumulation. However, for the cellulose powder, even after 168 hours of platinum addition, no platinum peaks were observed in XRF analysis and almost no platinum was trapped in the cellulose powder.

As described above, platinum was taken up by the *in vivo* samples, which were living algal cells, but the rate of uptake was very slow and platinum concentrations were still increasing 72–168 hours after addition. Such a slow uptake of platinum has also been reported as platinum accumulation in *G. sulphuraria*.^16^ In *G. sulphuraria* and other algae, cells rapidly uptake gold and silver.^23,24^ Platinum's unique behavior in terms of uptake time distinguishes it from other metals, making it a significant finding for metal recovery and biomining applications. In contrast, for the first time in this study, protoplasts with the cell wall removed were found to accumulate platinum at approximately twice the *in vivo* concentration. This suggests that the cell wall may prevent Pt uptake. Although cellulose is the main component of the cell wall, cellulose powder alone showed little trapping of Pt.

On the other hand, when the platinum solution was added to the lyophilized samples, Pt accumulated at higher concentrations than in living cells. This suggests that Pt accumulates in algal cells regardless of whether they are alive or dead. In the case of lyophilized samples, Pt accumulated at considerably higher concentrations than in live cell samples. If the presence of the cell wall inhibited Pt uptake, the high accumulation of Pt in lyophilized samples could be due to physical damage to the cell wall caused by the lyophilization process. Although no cell wall damage was visible in the SEM (scanning electron microscopy) image of the lyophilized samples, it is suspected that some damage to the cell wall occurred during lyophilized processes.

Platinum was also highly accumulated in the acetone-extraction residue samples. This suggests that the soluble components extracted with acetone, chlorophyll, lipids, and other biological compounds have little effect on the accumulation of platinum in algae and that some component (other than cellulose) in the residue was responsible for the high accumulation of platinum.

When the effect of light was examined, the concentration of platinum was higher in the light-exposed samples than in the light-shielded samples at all the addition times. Under light irradiation conditions, the samples that accumulated the highest amount of platinum among the samples prepared in this study were the lyophilized samples and the acetone-treated residue samples. Both samples accumulated the same amount of platinum and after 72 hours of addition, the platinum concentration was around 15 000 ppm. In the acetone-treated residue, soluble lipids and other substances were removed by the acetone treatment, but the absence of these substances may not have much effect on the high accumulation of platinum. The impact of light on platinum accumulation is intriguing. In living cells, platinum accumulation may be linked to metabolic factors. Conversely, even in dead cells (lyophilized or acetone-treated residues), light irradiation affects intracellular biomaterials and cell membranes, which may contribute to the high levels of platinum accumulation. The experiment was conducted in an incubator under constant temperature conditions, and light irradiation did not cause any temperature change.

It has been reported in the literature that the temperature at which platinum solution is added affects the removal of platinum from the solution by algae.^16^ The removal efficiency from the solution was higher at 40°C than at 4°C. Therefore, further research should be conducted to thoroughly examine the impact of temperature on the absorption of platinum by algae.

### Platinum distributions in the *in vivo* samples and the lyophilized samples

**Fig. 3:**
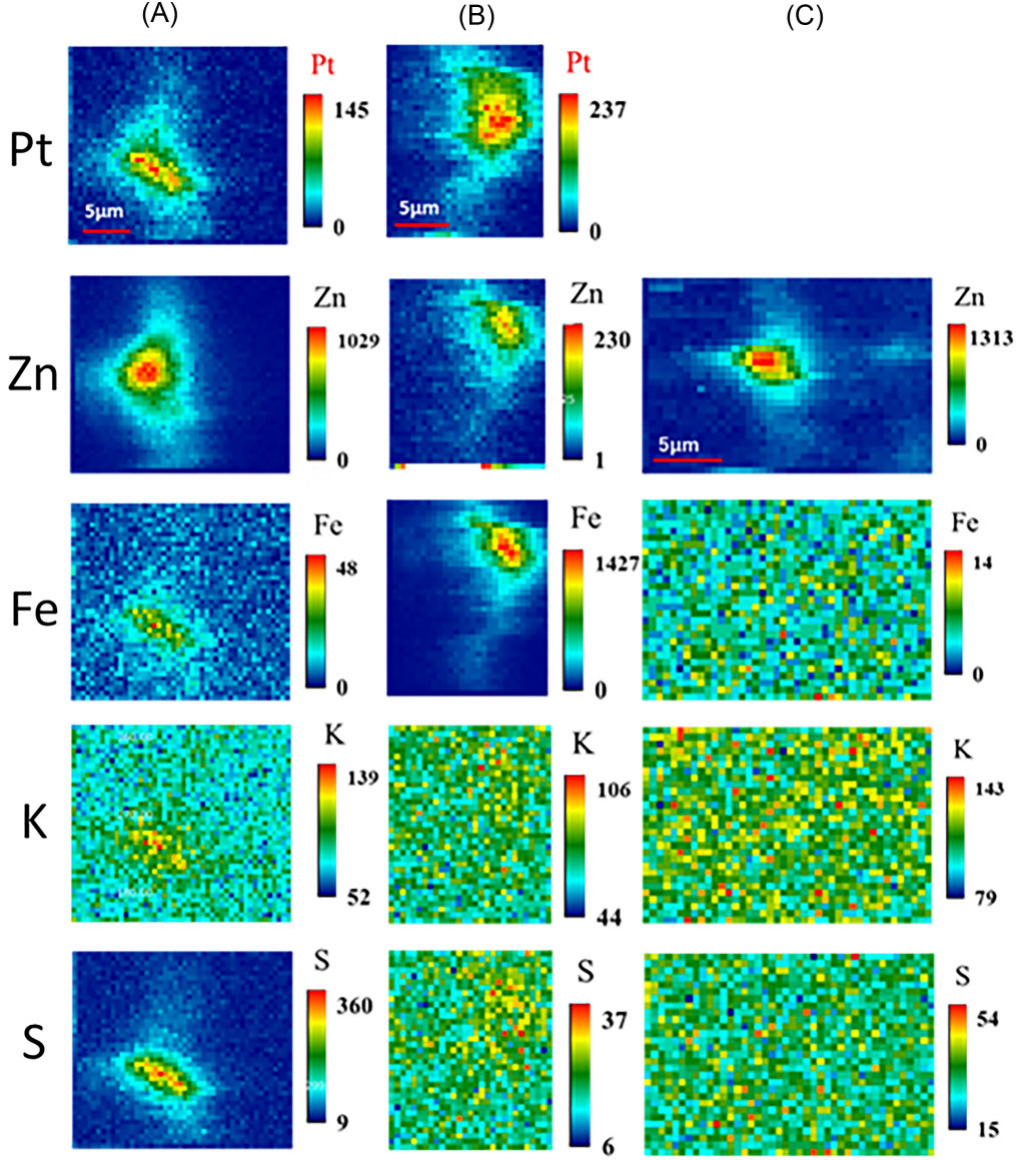
Elemental mapping in unicellular alga obtained by synchrotron radiation X-ray microbeam. (A) *In vivo* cell with platinum addition, (B) lyophilized cell with platinum addition, and (C) control cell. The concentration of the platinum solution added was 100 ppm, and the addition time was 24 hours, under light-shielded conditions. X-ray energy; 12.5 keV (A and B), 10 keV (C). X-ray beam size; 0.8 μm (V)×1 μm (H), step size; 0.5 μm (V)×0.5 μm (H), and measurement time; 2 s/point. Imaging area 23 μm(V)×20.5 μm(H)(A), 14.5 μm(V)×17.5 μm(H)(B), and 21 μm(V)×14.5 μm(H)(C).

The μ-XRF imaging results of the *in vivo* single cell, the lyophilized single cell, and the control live single cell without platinum addition are shown in Fig. 3(A)–(C). Due to the small size of the algal cells of *P. simplex*, μ-XRF imaging was performed by scanning an approximate area with a coarse 2D scan to detect signals from the cell, followed by a fine step size (0.5 μm) scan where the cell was present to obtain cell-level elemental distributions. In both the live cell sample and the lyophilized cell sample, platinum was added under light-shading conditions. Here, all samples were quick-frozen on sample plates and μ-XRF imaging was performed under cryogenic N_2_ gas flow. In other words, the results in Fig. 3 show non-destructively visualized elemental distributions while maintaining a water content close to that of living organisms. For the *in vivo* cell and the lyophilized cell in Fig. 3A and B, the incident X-ray energy was set to 12.5 keV to efficiently excite Pt L-lines. For the control cell sample, the incident X-ray energy was set to 10.0 keV. μ-XRF is a technique used to observe elemental distribution in cells. Here, we focused on the distribution of essential elements, particularly zinc, since bulk analysis has shown that the control does not contain platinum. To efficiently excite the Kα of Zn (8.62 keV), we selected 10 keV.

The spatial distribution of Zn detected in the control sample (Fig. 3C) is approximately 5 μm, which corresponds to the spatial distribution of a single cell. The algal cell of *P. simplex* has an elongated oval shape, so the cell center is thicker than the cell edge. The higher intensity of zinc near the center of the cell in the two-dimensional elemental distribution map may be due to the cell thickness. For iron, potassium, and sulfur, the XRF was not sensitive enough to obtain intracellular elemental distributions.

Zinc, an essential trace element, was detected in both the live cell (Fig. 3A) and the lyophilized cell (Fig. 3B), suggesting its intracellular presence. Moreover, Pt distribution throughout the cell, with higher X-ray fluorescence intensity at the center of both cell types (A for live cell, B for lyophilized cell), indicating the incorporation of Pt. Figure 3A and B demonstrate that platinum was not concentrated near the cell wall but distributed throughout the cytoplasm within the cell. Due to differences in cells between Figure 3A and B, it is challenging to compare platinum intensities. However, the lyophilized cell shows considerably higher Pt intensities, which is consistent with Table 1 results indicating that lyophilized samples accumulate more platinum than the *in vivo* samples.

Results for samples in which platinum was added during light irradiation are displayed in Fig. S1 in Supplementary data. Results for *in vivo* and lyophilized cells treated with platinum solution are presented in Panels (A) and (B), respectively. Both results were obtained by freezing cells with water content, and clearly indicate platinum incorporation into the cells.

For reference, Fig. S2 in the Supplementary data shows SEM observations of the algal cells containing platinum. After the platinum solution was added to the lyophilized sample, the sample was washed with pure water and subjected to lyophilization again before SEM analysis. The SEM image (a) in Fig. S2 shows that the cells retain their elongated oval shape. The EDS (energy dispersive X-ray spectroscopy) spectra of the cells (c) show that the essential elements such as C, N, O, Mg, P, S, Cl, and K were detected. The elemental mapping is also shown in (b). The distribution of P was clearly visible inside the cell. The distribution of phosphorus seen here is considered poly-P. Poly-P is a biological polymer composed of inorganic phosphate residues linked by ‘high-energy’ phosphoanhydride bonds. Poly-P has a wide range of regulatory functions, including roles as energy and inorganic phosphate reservoirs, and is found in unicellular algae such as *Chlamydomonas reinhardtii, Parachlorella kessleri*, and *Chlorella* species.^25^

### The oxidation state of platinum in algal samples estimated from Pt L_3_ spectra

The XANES analysis is an effective technique for evaluating an oxidation state. The oxidation number of arsenic and manganese compounds can be determined by analyzing the energy shift of the peak in the XANES spectrum.^26,27^ However, for platinum, the energy shift of the peak is minimal, making it challenging to determine the oxidation number based on this factor. Previous research indicates that the white line intensity, i.e. the peak height of the absorption edge, of the L_3_ absorption edge XANES spectrum of 5d transition elements is dependent on the number of electrons in the 5d orbital.^20^ The white line represents a tendency to increase as the oxidation number of platinum in a platinum compound increases, corresponding to a decrease in the packing of the 5d orbitals, which can be used to distinguish platinum of different valences. Previous reports have shown L_3_ spectra of platinum during the synthesis of platinum nanoparticles in solution, which clearly display the transition from a tetravalent platinum ion to a zero-valent metal.^28^ To verify this, we conducted measurement of Pt L_3_-edge XANES spectra using reference materials with established chemical forms. Figure S3 shows the Pt L_3_ edge XANES spectra of the reference materials such as Pt foil, PtCl_2_, and H_2_PtCl_6_ as an example. The absorption (μt) is plotted on the vertical axis, normalized to 1.0 at 11 585 eV. The white line energy is identified by the absorbance peak height at 11 566 eV.

Table 2 provides a summary of the peak heights for the white line obtained from each material, the oxidation number of platinum, and the number of electrons in the 5d orbitals in each material. The Pt foil has an oxidation number of zero, 9 electrons in the 5d orbital, and a peak height of 1.32. On the other hand, the platinum in the hexachloroplatinate(IV) solution used for algae addition has an oxidation number of +4 and the number of electrons in the 5d orbital is 4. The peak height in the hexachloroplatinate(IV) solution was 2.46. As seen in Table 2, the higher valence (oxidation number) of platinum indicates the higher intensity of the white line at the L_3_ absorption edge.

**Table 2:** Normalized peak heights obtained from the platinum reference materials of various valence

Reference material	Number of electrons in 5d orbitals	Valence	Normalized peak height^a^
Pt foil	9	0	1.32
PtCl_2_	6	+2	1.45
Cisplatin	6	+2	1.45
H_2_PtCl_6_ (solution)	4	+4	2.46
H_2_PtCl_6_ (solid powder)	4	+4	2.42
PtO_2_	4	+4	2.41

^a^The peak height of each spectrum obtained by normalizing the absorbance (μt) at 11 585.523 eV of the spectrum to be 1.0.

**Fig. 4:**
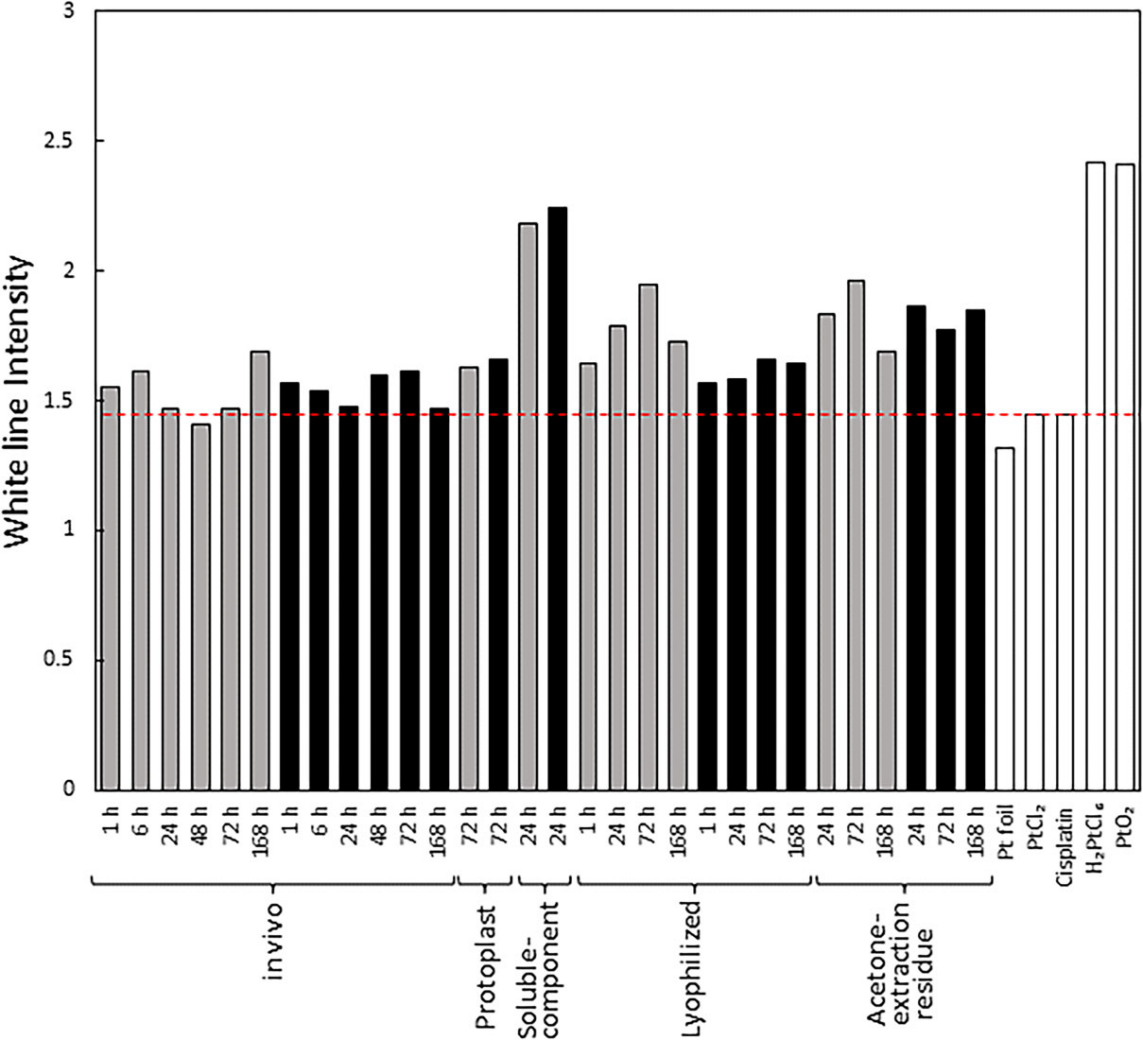
White line intensity of platinum L_3_-edge XANES spectrum obtained from the algal samples and platinum reference materials (gray bar; light irradiation, black bar; light shading, and white bar; reference materials). The white line intensity was obtained from 11 566 eV. The dashed line is a guide for +2 valence platinum compounds. The concentration of the platinum solution added was 100 ppm.

The Pt XANES spectra for *in vivo* cells and the soluble component fraction of algae treated with hexachloroplatinate(IV) are shown as an example in Fig. S3. The absorbance peak intensity at 11 566 eV was identified as the peak height of the white line. The peak intensities of the white line in various algal samples with hexachloroplatinate(IV) solution are identified and presented in Fig. 4 alongside the reference materials. As XANES spectra are additive, the height of the white line indicates the average oxidation state of platinum compounds present in the sample.

The white line peak heights for *in vivo* samples where Pt was added with illumination varied from 1.41 to 1.69 at all addition times ranging from 1 to 168 hours post-addition, aligning closely with the peak height (1.45) of reference materials possessing an oxidation number of +2. The peak heights for *in vivo* samples added to the platinum solution under light-shielded conditions were comparable to those of the illuminated samples. The solution of hexachloroplatinate(IV) ion was introduced to the algae. The oxidation number of platinum in H_2_PtCl_6_ is +4, whereas the platinum integrated into the algae was brought down from +4 to +2 valence. It is inferred that platinum included in the algae was not reduced to the 0-valent metallic state since the height of the white line peak did not reduce to the Pt foil's height (1.32) under both light–light and light–shadow conditions.

One hour after adding platinum, the white line's peak height in the *in vivo* sample was 1.55, which is lower than the hexachloroplatinate(IV)’s peak height (2.46) in the solution. Even after 168 hours, there was little change in this peak height. The algae's platinum concentration increased slowly (see Table 1), while the reduction reaction of platinum proceeded relatively quickly.

The peak heights from the XANES spectra of the protoplast samples, taken 72 hours after the addition, were 1.63 and 1.66 for the illuminated and shaded samples, respectively. The values were slightly higher than the peak height of platinum references (PtCl_2_ and cisplatin) with an oxidation number +2 (1.45), but lower than that of hexachloroplatinate ion (2.46). The peak heights of *in vivo* samples ranging from 1.41 to 1.69, which are comparable to the obtained results. Therefore, it can be concluded that Pt accumulated in protoplasts was mostly reduced.

In the samples where platinum solution was added to the soluble component fraction from crushed cells, the obtained peak heights were higher (1.69–1.96) compared to *in vivo* and lyophilized samples and were lower than those of platinum compounds having an oxidation number of +4 (2.46). This implies that Pt was not significantly reduced by the soluble component alone.

The peak height of the Pt-added samples in the lyophilized state was comparable under light-shielded conditions to that of the *in vivo* samples and +2-valent Pt compounds. Additionally, the peak height of the samples with platinum added under light collection was slightly higher than that of the shaded samples.

The peak heights acquired from the residue treated with acetone were marginally higher compared to those of the *in vivo* and lyophilized samples. This suggests that the residue samples treated with acetone contained more platinum compounds that were not reduced.

Thus, of the algae samples prepared in the experiments, the *in vivo* samples typically showed the lowest peak height of the white line, which indicates that the chemical species reduced to +2 valence were especially abundant in the *in vivo* samples. Despite the low platinum accumulation in the *in vivo* samples, the physiological functions of the living algal cells suggest that they may have a positive involvement in the reduction of platinum. However, it is difficult to distinguish between intermediate oxidation states and mixtures of multiple oxidation numbers based on XANES spectra alone. Therefore, we also investigated the EXAFS analysis of the L_3_ absorption edge of platinum.

### EXAFS analysis of Pt L_3_ absorption edge

**Fig. 5:**
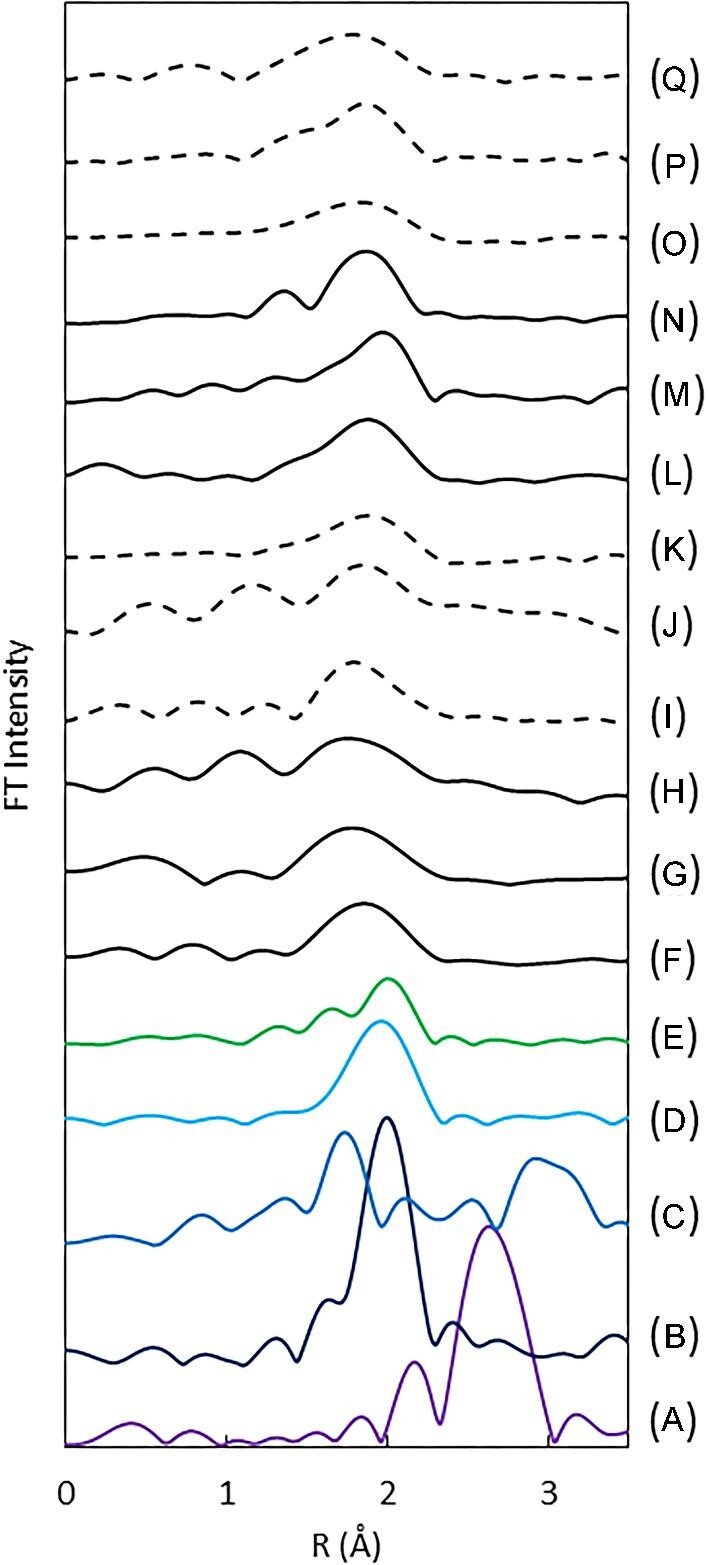
Radial structure function of Pt obtained from EXAFS analysis of Pt-addition algae samples and platinum reference materials. (A) Pt foil, (B) H_2_PtCl_6_, (C) PtO_2_, (D) PtCl_2_, (E) cisplatin (*cis*-diamminedichloro-platinum), (F)–(K) *in vivo* samples, and (L)–(Q) lyophilized samples. (F)–(H) and (L)–(N) were under light irradiation during Pt addition. (I)–(K) and (O)–(Q) were under light shading during Pt addition. The Pt addition time was 24 hours (F), (J), (L), and (O), 72 hours (G), (J), (M), and (P), and 168 hours (H), (K), (N), and (Q).

The analysis of EXAFS oscillation in X-ray absorption spectra provides crucial information regarding the local structure of the element of interest, including the atoms coordinated to the element, coordination distance, and coordination number. Figure S5 of Supplementary data presented the *k*^3^  *χ*(*k*) oscillations of EXAFS spectra for Pt L_3_-edge, displaying the data for both the algal samples and reference samples, such as H_2_PtCl_6_, Pt foil, PtO_2_, PtCl_2_, and cisplatin. We analyzed the oscillation of EXAFS in Pt L_3_-edge spectra obtained from *in vivo* and lyophilized samples to which hexachloroplatinate(IV) was added. Figure 5 shows the FT data for the *k*^3^-weighted *χ*(*k*) values for Pt. The phase shift was not corrected in the FT. The highest peak in the FT corresponds to the Pt-ligand interaction of the nearest coordination sphere. The strongest peaks of the reference materials, specifically H_2_PtCl_6_ (Fig. 5B) and PtCl_2_ (Fig. 5D), correspond to the Pt–Cl bond. Meanwhile, the peaks of PtO_2_ (Fig. 5C) and Pt foil (Fig. 5A) correspond to the Pt–O bond and Pt–Pt bond, respectively. Cisplatin (Fig. 5E), which has both nitrogen and chlorine atoms coordinated to a platinum atom, exhibits two peaks. Our work involved curve-fitting analysis to obtain structural parameters for platinum reference materials. Curve-fitting analysis was performed on the radial structure functions of the reference substances, following the fitting of the first strongest peak displayed in Fig. 5. Table 3 shows the calculated coordination numbers and bond lengths for the reference materials.

**Table 3:** Results of Pt L_3_-EXAFS curve-fitting analysis for the first shell

Sample	ligand	*N*	*r* (Å)	*σ* ^2^ (Å^2^)	*R* (%)
Pt foil	Pt	12	2.76(2.77)^29^(2.762)^30^	0.07	2.075
H_2_PtCl_6_(solid powder)	Cl	5.58	2.32(2.32)^29^(2.314)^30^	0.04	0.715
PtO_2_	O	3.22	2.04(2.00)^29^(2.010)^30^	0.022	0.307
PtCl_2_	Cl	4	2.32(2.32)^29^	0.067	1.015
cisplatin	N	2	2.08(2.052)^31^	0.049	0.485
	Cl	2	2.31(2.330)^31^	0.051	

*N*: coordination number; *r*: bond length; *σ*: Debye–Waller factor; and *R*: reliability factor for goodness of fit, which was calculated as

${R}^2 = \ \displaystyle\frac{{\sum {{\{ {{k}^3{\chi }_{obs}(k) - {k}^3{\chi }_{{calc}}(k)} \}}}^2}}{{\sum {{\{ {{k}^3{\chi }_{obs}(k)} \}}}^2}} \times 100$

The parenthesized values are reported in the references.

The literature reports the Pt–Pt bond distance in platinum metal crystals as 2.77^29^ or 2.762 Å.^30^ The Pt–Cl bond distance of hexachloroplatinate(IV) was 2.32^29^ or 2.314 Å,^30^ while that of platinum(II) chloride measured 2.30 Å.^30^ Additionally, the Pt–O bond distance of platinum(IV) oxide measured 2.00^29^ or 2.010 Å.^30^ The Pt–N binding in cisplatin was reported as 2.052 Å, while the Pt–Cl was found to be 2.330 Å.^31^ Our analytical results for the reference materials were consistent with these literature values, as displayed in Table 3. Since the platinum of cisplatin is coordinated with both the nitrogen and chlorine atoms, these bonding distances were determined by fitting them together.

The results of *in vivo* cells collected between 6 and 168 hours after adding platinum are displayed in Fig. 5(F)–(K). The outcomes of light irradiation and light shielding are specifically indicated by (F)–(H) and (I)–(K), respectively. If platinum were taken up in its added chemical form, a distinct Pt–Cl bond peak (Fig. 5B) would be visible due to the excellent symmetry of the hexachloroplatinate(VI) complex ion, since the chemical species added to the algae was H_2_PtCl_6_. However, in samples (F)–(K), the peak intensity derived from the first coordination sphere of platinum was low and the peak width was broad.

The strongest peak obtained from the algae samples (F)–(K) demonstrated a shorter binding distance than the Pt(IV)–Cl bond peak (Fig. 5B) and the Pt(II)–Cl bond peak (Fig. 5D), as indicated by the *r*-value. The *r*-values exhibited by the algae samples were longer than the peak of the platinum–nitrogen bond distance (Fig. 5E) and shorter than the platinum(IV)–chlorine bond distance (Fig. 5B). This suggests that the platinum of the added hexachloroplatinate(IV) changed to a chemical species bound to a different ligand in the algal cells where it was taken up. Comparable outcomes were observed when platinum was added to lyophilized cells, as reported in Fig. 5(L)–(Q).

Comparing the *in vivo* samples (F)–(H) and the lyophilized samples (L)–(N) with platinum added under light irradiation, the *r* values of the peaks were slightly shorter in the *in vivo* samples. Conversely, when platinum was added without light irradiation, there was no significant difference between *in vivo* samples (I)–(K) and lyophilized samples (O)–(Q).

It is difficult to determine the speciation of platinum incorporated into the cells solely from the peak fitting results obtained here. However, fitting the FT values of the EXAFS vibrations indicates that the platinum incorporated into the algae has been changed to a chemical species with Pt–N or Pt–O bonds, with shorter bonds than the original Pt–Cl bonds in H_2_PtCl_6_.

The platinum form found in *G. sulphuraria*, a type of hot spring algae, has been identified as a platinum complex coordinated to sulfur and designated as [(C_2_H_5_)_2_S]PtCl_2_. However, the EXAFS analysis of platinum in *P. simplex* treated in this study did not yield a definite peak for Pt–S binding (2.303 Å^30^ in platinum sulfide, PtS). While hot spring algae can survive in low pH environments containing hydrogen sulfide, the cells investigated in this research were freshwater aerobic algae. The chemical form of platinum accumulated in *P. simplex* was not a sulfur coordination complex in this study, signifying potential variations among algal species. The EXAFS analysis of platinum in algae did not reveal any peaks originating from Pt–Pt, indicating that platinum in the metallic state did not coexist. As platinum's stable oxidation states are +4, +2, and 0, it is difficult to assume an intermediate state of +3. In Fig. 4, some samples have a lower white line intensity compared to the added hexachloroplatinate(IV). This was probably due to the presence of a reduced state with an oxidation number of +2 and not to the coexistence of metallic platinum (oxidation number 0).

### Mechanism of platinum accumulation in the unicellular alga *P. simplex*

The uptake of heavy metals into plant cells is thought to proceed in three steps: (1) contact with the cell surface, (2) adsorption to the cell surface, and (3) uptake into the cell interior. If the interaction between the cell surface and the metal ion is weak, it can be easily removed by cell washing. On the other hand, the cell surface contains polysaccharides, proteins, and complex lipids, some of which may bind metal ions. The adsorption process is influenced by various factors, including ion size, charge density, and other substrate-related factors.^32,33^

This study utilized micro-XRF imaging to observe that platinum accumulated within algal cells instead of on their surfaces. On the other hand, EXAFS analysis revealed that adding platinum to algae in the form of hexachloroplatinate(IV) altered its chemical structure within the cells where it was stored. Although the rate of platinum accumulation was quite slow, the chemical form of the platinum incorporated into the algae remained largely unchanged after either 24 or 168 hours. The XANES analysis of the Pt L_3_-edge indicated that the majority of platinum in the *in vivo* and the protoplast samples were reduced from a valence state of +4 to +2. However, in the lyophilized samples, the peak heights of the Pt L_3_-edge were slightly higher than that of the +2-valence reference materials, suggesting some presence of +4-valent compounds in the mixture. The reduction occurred in protoplasts but at a lower rate in the soluble component fraction and the acetone-extraction residue. This suggests that the platinum reduction mechanism was inhibited in the disrupted cellular state.

The slow rate of platinum accumulation indicates a change in the chemical form of the Pt complex during uptake by algal cells. It is possible that the Pt–Cl bonds in the hexachloroplatinate ions were gradually substituted with Pt–N or Pt–O bonds, resulting in new chemical species that were taken up through the cell membrane. Considering that the valence of Pt integrated into the algae remains constant over time, the ratio of platinum complexes on the cell surface to those within the cell is almost steady. This was also observed in a prior study on platinum accumulation by *C. stigmatophora*, a marine microalga, where the ratio of surface-absorbed platinum to that incorporated into the algae remained constant over time.^12^ If the ligand exchange reaction proceeds rapidly and replaces the Pt–Cl bonds with Pt–N/O bonds, the accumulation of Pt in the algal cell may also occur rapidly.

The effect of light on Pt uptake was investigated, and a tendency for more Pt to accumulate was observed with light irradiation. This finding is particularly interesting as it suggests that Pt accumulation in living cells may be influenced by metabolic factors. Even in dead cells (lyophilized or acetone-treated residues), light irradiation affected platinum accumulation, indicating that this phenomenon could be both biological and chemical in nature. The chemical effect may be related to the fact that the ligand exchange reaction of the hexachloroplatinate(IV) complex is accelerated under light irradiation.^34^ No chemical changes were observed in the platinum solution alone, even under light irradiation conditions. Therefore, it is assumed that the reduction of Pt by cell-derived organic matter and the ligand exchange reaction of the platinum(IV) complexes promoted the light-induced uptake of Pt even in dead cells. During the reduction reaction, light irradiation can promote the reduction of Pt or the ligand exchange reaction. However, in this case, the reduction reaction proceeded without light irradiation, indicating that the biological compounds present in the algal cells, such as extracellular polymeric substance, acted as reducing agents instead of photo-reduction.

## Conclusions

The study examined the uptake of hexachloroplatinate(IV) in the unicellular alga, *P. simplex*, and found that the cells integrated primarily +2-valent chemical species of platinum. The highest percentage of +2-valent species was identified in the *in vivo* cells, the protoplasts, the lyophilized, the acetone-extraction residues, and the soluble component fraction, in sequential order. The percentages of +2 and +4 valences did not fluctuate considerably within the range of addition times, 1–168 hours.

Based on the results, we propose a scheme in which the Pt–Cl bond of hexachloroplatinate(IV) on the cell surface is slowly replaced by Pt–N or Pt–O bond. The ligand-exchanged platinum complex is then incorporated into the cell interior. Further studies are needed to validate this scheme rigorously.

In this study, we used synchrotron radiation X-ray analysis to measure intact algal cells without destroying them. The μ-XRF imaging measurement at SPring-8 enabled observation of a single cell holding water while broadly maintaining its vitality.

The method that utilized freeze-dried samples under light irradiation demonstrated the highest efficiency in platinum recovery. This is because the uptake of platinum was shown to proceed more slowly than that of gold or silver, allowing for differentiation in speed that can be utilized for metal recovery and biomining. The prospect of heavy metal recovery utilizing algae’s physiological function as an eco-friendly recycling technology is promising.

## Supplementary Material

mfae009_Supplemental_File

## Data Availability

The data underlying this article will be shared on reasonable request to the corresponding author.
