# Supplementary material for: Uptake and accumulation mechanisms of hexachloroplatinate(IV) ions in the unicellular alga, Pseudococcomyxa simplex
Source: Metallomics. 2024 Jan 31;16(2):mfae009. doi: 10.1093/mtomcs/mfae009 (PMC10858386; doi:10.1093/mtomcs/mfae009)
Supplement: mfae009_Supplemental_File [file mfae009_supplemental_file.docx]

**Uptake and accumulation mechanisms of hexachloroplatinate(IV) ions in the unicellular alga, *Pseudococcomyxa simplex***

Masato Tokoro ^a^, Yu Imamura ^a^ , Kazuhiro Kumagai ^b^, Akiko Hokura ^c*^

a Graduate School of Engineering, Tokyo Denki University, 5 Senju-Asahicho, Adachi, Tokyo 120-8551, Japan

b Nanodimensional Standards Group, Research Institute for Material and Chemical Measurement National Metrology Institute of Japan (NMIJ), National Institute of Advanced Industrial Science and Technology (AIST), Tsukuba Central 5, 1-1-1 Higashi Tsukuba, Ibaraki 305-8565

c Department of Applied Chemistry, 5 Senju-Asahicho, Adachi, Tokyo 120-8551, Japan

* Corresponding author

Table S1　 Composition of culture medium [17]

| Chemicals | Quantity(g/L) |
| --- | --- |
| MgSO_4_·7H_2_O | 25.0 |
| KH_2_PO_4_ | 25.0 |
| KNO_3_ | 101 |
| FeSO_4_·7H_2_O | 1.25 |
| CuSO_4_・·5H_2_O | 0.0550 |
| H_3_BO_3_ | 0.614 |
| CoCl_2_·6H_2_O | 0.0550 |
| NaMoO_4_·2H_2_O | 0.0250 |
| ZnSO_4_·7H_2_O | 0.0550 |
| MnSO_4_·5H_2_O | 0.390 |
| KI | 0.0280 |
| (NH_4_)_2_C_4_H_4_O_6_ | 9.20 |
| CaCl_2_·2H_2_O | 0.736 |


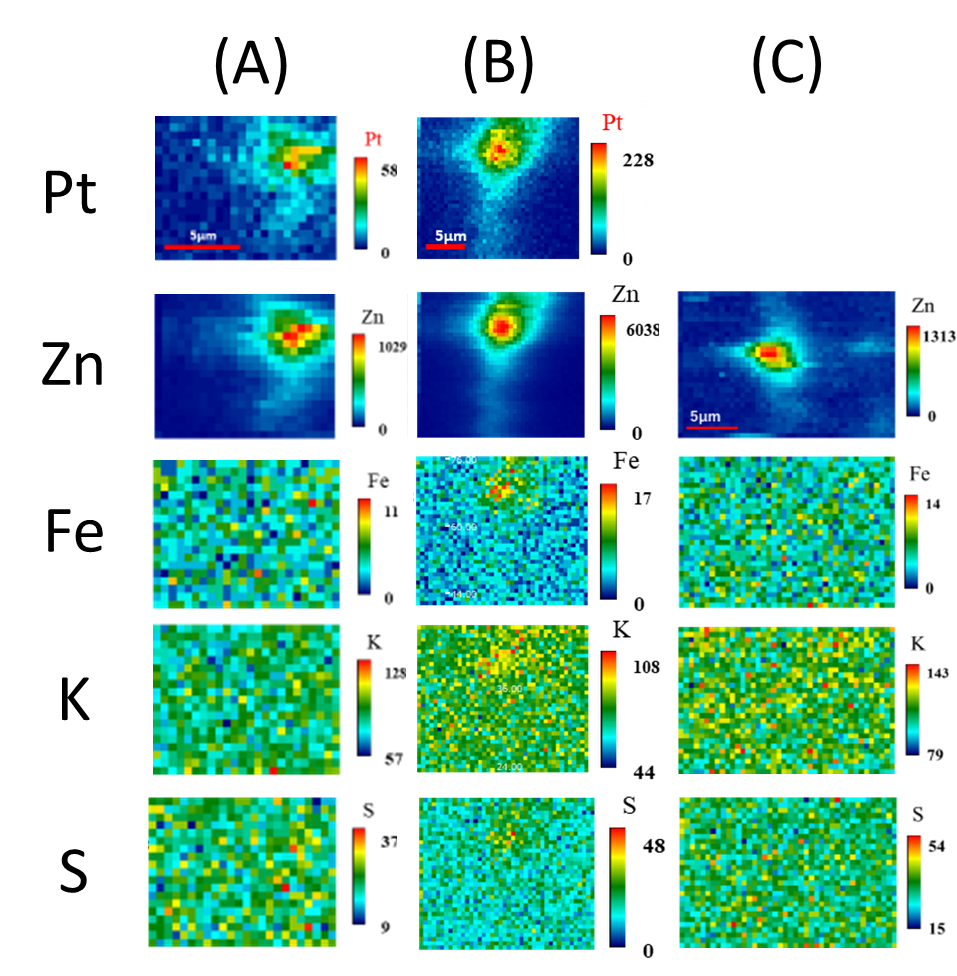


Figure S1　　Elemental mapping in unicellular alga obtained by synchrotron radiation X-ray microbeam

(A) in vivo cell with platinum addition and (B) lyophilized cell with platinum addition.

The concentration of the platinum solution added was 100 ppm, and the addition time was 24 hours, under light irradiation conditions.

X-ray energy; 12.5 keV (A and B).

X-ray beam size; 0.8 µm (V)×1 µm (H), step size; 0.5 µm (V)×0.5 µm (H), measurement time; 2 s/point. Imaging area 23 µm(V)×9.5 µm(H)(A)，21 µm(V)×18 µm(H)(B).


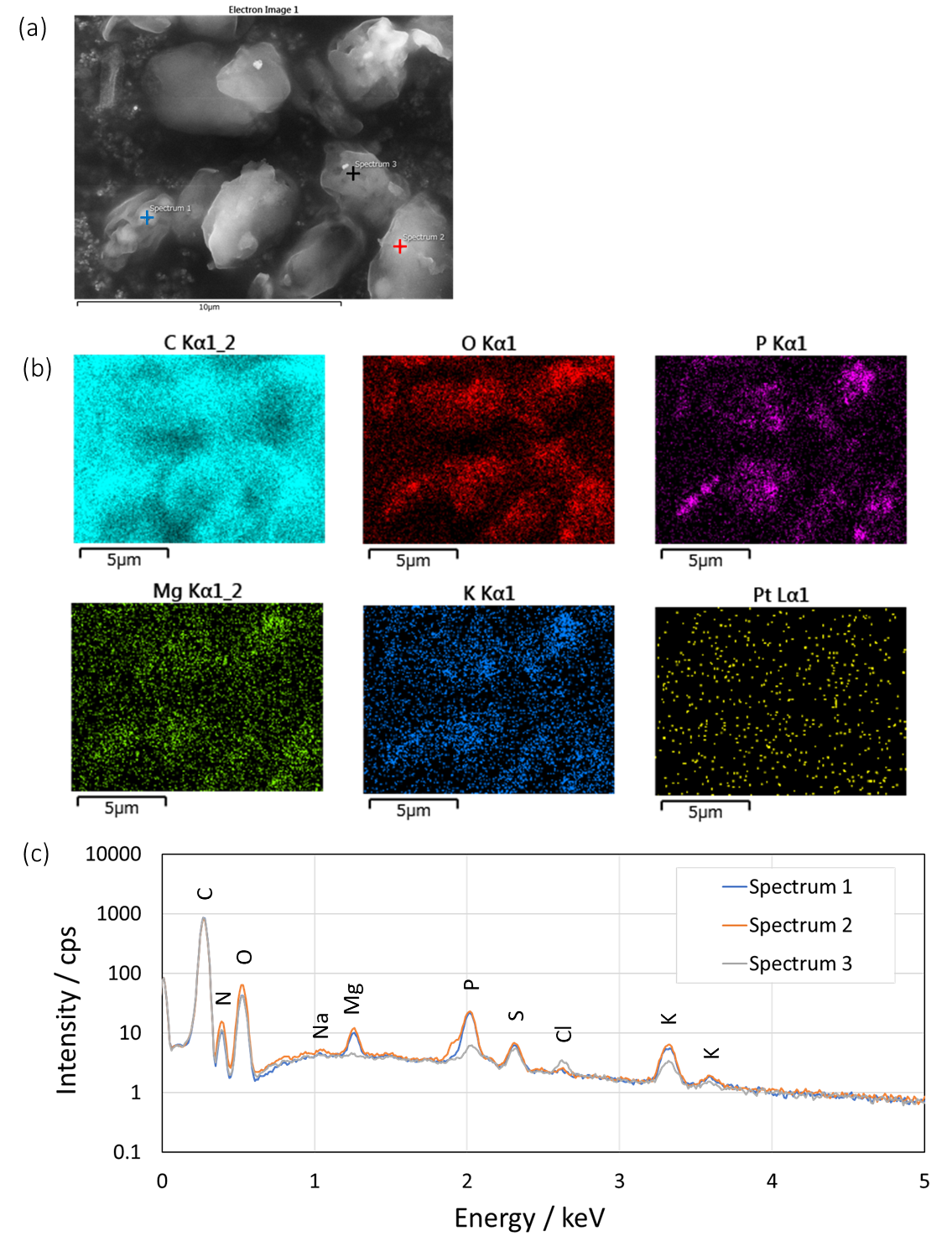


Figure S2　　SEM observation for the algal cells added with platinum

The lyophilized cells were shaken with platinum solution ([Pt] = 100 mg/kg) and under light irradiation for 168 hours. (a) SEM image, (b) elemental mapping, (c) EDS spectra.


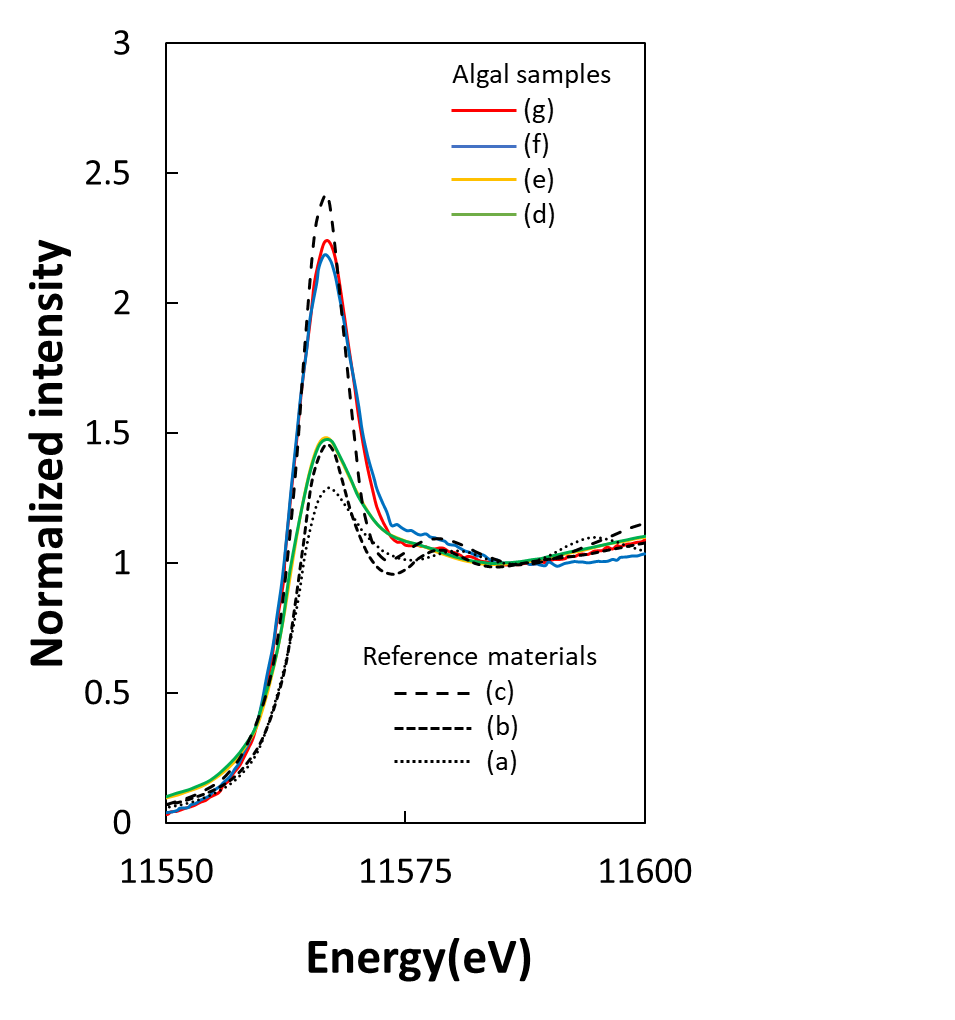


Fig. S3 Platinum L_3_-edge XANES spectra obtained from the algal samples added platinum and the reference materials.

(a) Pt foil, (b) PtCl_2_, (c) H_2_PtCl_6_, (d) in vivo cells with Pt under light irradiation, (e) in vivo cells with Pt under light shading, (f) extract sample with Pt under light irradiation, (g) extract sample with Pt under light shading.

The concentration of the platinum solution added was 100 ppm, and the addition time was 24 hours.
